# siRNA-Based Targeting of Cyclin E Overexpression Inhibits Breast Cancer Cell Growth and Suppresses Tumor Development in Breast Cancer Mouse Model

**DOI:** 10.1371/journal.pone.0012860

**Published:** 2010-09-20

**Authors:** Yulong Liang, Hong Gao, Shiaw-Yih Lin, John A. Goss, Francis C. Brunicardi, Kaiyi Li

**Affiliations:** 1 The Michael E. DeBakey Department of Surgery, Baylor College of Medicine, Houston, Texas, United States of America; 2 Department of Systems Biology, M.D. Anderson Cancer Center, Houston, Texas, United States of America; Universidade de São Paulo, Brazil

## Abstract

Cyclin E is aberrantly expressed in many types of cancer including breast cancer. High levels of the full length as well as the low molecular weight isoforms of cyclin E are associated with poor prognosis of breast cancer patients. Notably, cyclin E overexpression is also correlated with triple-negative basal-like breast cancers, which lack specific therapeutic targets. In this study, we used siRNA to target cyclin E overexpression and assessed its ability to suppress breast cancer growth in nude mice. Our results revealed that cyclin E siRNA could effectively inhibit overexpression of both full length and low molecular weight isoforms of cyclin E. We found that depletion of cyclin E promoted apoptosis of cyclin E-overexpressing cells and blocked their proliferation and transformation phenotypes. Significantly, we further demonstrated that administration of cyclin E siRNA could inhibit breast tumor growth in nude mice. In addition, we found that cyclin E siRNA synergistically enhanced the cell killing effects of doxorubicin in cell culture and this combination greatly suppressed the tumor growth in mice. In conclusion, our results indicate that cyclin E, which is overexpressed in 30% of breast cancer, may serve as a novel and effective therapeutic target. More importantly, our study clearly demonstrates a very promising therapeutic potential of cyclin E siRNA for treating the cyclin E-overexpressing breast cancers, including the very malignant triple-negative breast cancers.

## Introduction

Cyclin E (cycE), encoded by *cyclin E/CCNE1*, is an important cell cycle regulator, which promotes G1/S transition by activation of Cdk2 kinase activity [1], [2]. CycE expression in normal dividing cells is upregulated at late G1 phase by transcription activation through E2F family transactivators [3]. The accumulated cycE at G1/S boundary simultaneously forms complex with Cdk2 and subsequently promotes initiation of DNA replication and centrosome duplication. The abundant cycE eventually becomes phosphorylated and destroyed by ubiquitin-mediated proteolysis that allows normal cell cycle progression [4]–[7]. However, the level of cycE and activity of cycE-Cdk2 can be aberrantly regulated and this excessive activity of the cycE-Cdk2 complex, in turn, drives cells to replicate their DNA prematurely, resulting in genome instability [8], [9] and tumorigenesis [10]. In breast cancer, cycE is overexpressed in ∼30% patients [11], including overexpression of both the full length (50 kDa) and several low molecular weight (LMW) isoforms (ranging in size from 33 to 45 kD) of cycE protein [12], [13]. Importantly, total levels of cycE (both full length and LMW isoforms) in tumor tissues are inversely correlated with survival in patients with breast cancer [14]. The patients whose cancers show high levels of cycE at stage I die within five years of diagnosis, while in contrast, cycE-low expressing patients have a much longer survival, indicating that overexpressed cycE may be an important cause for breast cancer mortality, and cycE may serve as an important therapeutic target for the development of anticancer drugs.

RNA interference (RNAi) is a mechanism for RNA-guided regulation of gene expression in which double-stranded ribonucleic acid (dsRNA) results in rapid destruction of mRNA containing the identical sequence as the dsRNA. The functional mediator of RNAi is ∼21-nt siRNAs (small interfering RNAs) generated by cleavage of dsRNAs via a complex consisting of Dicer, TAR RNA-binding protein (TRBP) and protein activator of protein kinase PKR (PACT) [15]–[17]. In fact, in the past several years, chemically synthesized siRNA oligos have been proven to be superior agents that can effectively knockdown gene expression by sequence-specific degradation of its complementary mRNA in cell culture. As compared to conventional antisense oligonucleotide approach, siRNA-mediated gene knockdown is much more specific and potent. The selection of the targeting sequences of siRNA is less restricted so the rates of producing effective duplexes are higher [18]. In addition, siRNA is double stranded RNA, which is more resistant to nuclease degradation, and therefore it can have prolonged stability in *in vivo* studies [19], [20]. These unique properties make siRNAs a promising new class of drugs for cancer treatment via targeting the mutation- or overexpression-activated oncogenes in cancers. Numerous recent studies have shown that siRNA can effectively suppress oncogene expression in cancer cells [17], [21]–[23] and a couple of siRNA cancer therapies are indeed in preclinical or early-stage of clinic trials [17]. In this study, to investigate if cycE can serve as a novel therapeutic target and if siRNA-based approach can effectively treat cycE-overexpressing breast cancer, we employed cycE siRNA to target cycE overexpression and assessed its ability to suppress breast cancer growth in nude mice. Our study here clearly demonstrated a very promising therapeutic potential of cycE siRNA for treatment of cycE-overexpressing breast cancer, including the highly malignant triple-negative breast cancer.

## Methods

### Ethics statement

All animal protocol performed in this study was approved by the Institutional Animal Care and Use Committee at Baylor College of Medicine (protocol number: AN-3142) and nude mice aged 8–12 weeks were used for in vivo studies.

### Cell culture

All cell lines used here were obtained from ATCC (Rockville, MD) and cultured at 37°C in a 5% CO2 incubator. Immortalized normal human mammary epithelial cell line MCF-10A was cultured in DMEM/F12 with 5% horse serum (Invitrogen), 10 µg/ml insulin, 20 ng/ml EGF, 0.5 µg/ml hydrocortisone and 1% penicillin/streptomycin (Invitrogen). Breast cancer cell lines including basal type MDA-MB436 (ER-, PR-negative) and MDA-MB157 (ER-, PR-negative), and luminal type SK-BR3 (ER-, PR-negative, HER2-overexpressed), MDA-MB453 (ER-, PR-negative) and T47D (ER-, PR-positive) [24] were cultured in DMEM supplemented with 10% fetal bovine serum (Invitrogen), 1% L-glutamine (Invitrogen) and 1% penicillin/streptomycin (Invitrogen), while for MDA-MB436, 10 µg/ml insulin was also added into the above medium.

### Transfection with siRNA oligos

The siRNA oligos for cyclin E (cycE) and luciferase (Luc) were synthesized by Dharmacon Research Inc. The cycE siRNA oligos corresponded to nucleotides 592 to 610 of the human *CCNE1* (variant 1) coding region (GenBank accession number: NM_001238). The indicated breast cancer cells (1×10^5^/well) were transfected with siRNA oligos (0.3 µg/well) in 6-well plates using Oligofectamine reagent (Invitrogen) following the manufacturer's protocol.

### Western blotting analysis

Forty four hours post-transfection, cells were lysed, as indicated, into mammalian cell lysis buffer (20 mM Tris-HCl pH 7.5, 150 mM NaCl, 0.5% NP-40, 1 mM EDTA, 1 mM EGTA, 1 mM DTT) with 5 mM sodium fluoride, 1 mM sodium orthovanadate, 1 mM phenylmethyl sulfonylflouride, 2 µg/ml aprotinin, 2 µg/ml leupeptin. After centrifugation at 4°C (14,000 rpm, 15 min), lysates (20 µg) were analyzed by immunoblotting assay. Anti-cycE polyclonal antibody (C-19) was from Santa Cruz and anti-actin antibody (Ab-1) was from Oncogene Research (Boston, MA). The densities were determined by densitometry for each protein band, and the density of cycE was standardized against that of actin in each sample. The standardized density of cycE in the mock SK-BR3 was arbitrarily set at 100%, and the relative level of cycE in other samples was obtained by comparing those standardized densities of cycE to that of cycE in the mock SK-BR3. The data shown here were presented as means ± s.d. from at least three independent experiments.

### Apoptosis and cell cycle analysis

Standard fluorescence-activated cell sorter (FACS) analysis was used to determine apoptosis of the cells or the distribution of the cells in cell cycle. Briefly, the cells were transfected with or without siRNA. Adherent cells were then collected by trypsinization and combined with cells floating in the medium. The cell cycle pattern was analyzed after being stained with propidium iodide, and the apoptotic cells were simultaneously assessed by flow cytometric detection of sub-G1 DNA content.

### Colony formation assay in soft agar

The standard colony formation assay was performed as described previously [25]. Briefly, the indicated breast cancer cells were transfected without (mock) or with siRNA oligos targeting cyclin E (cycE) or luciferase (Luc). Two days after transfection, the cells (1×10^3^ cells/well) were plated in 24-well plates in culture medium containing 0.35% agar overlying a 0.7% agar bottom layer and cultured at 37°C with 5% CO_2_. Three to five weeks later, the top layer of the culture was stained with *p*-iodonitrotetrazolium (1 mg/ml), and colonies (>100 µm) in diameter were counted. To monitor the cell viability of each group, the cells (1×10^3^ cells/well) were also plated in the common 10-cm culture plates with the normal complete medium, and 2–3 weeks later, the colonies were stained with 1% crystal violet, and counted. The number of colonies in soft agar was normalized by the cell viability of each group. Finally, the normalized number of colonies in soft agar for the groups of Luc or cycE was standardized against the control cells (mock, set at 100%).

### *In vivo* tumor studies

MDA-MB436 (1∼2×10^6^/injected point), SK-BR3 (1∼2×10^6^/injected point) or T47D (1∼2×10^6^/injected point) mixed with Matrigel Matrix (BD Biosciences) were injected into the second pair of the mammary glands of the nude mice (two injected points per mouse, and 45 mice for each cell line). Twelve days or four weeks later, the injected nude mice with tumor burden were randomly divided into 3 groups (n = 15 for each group): mock (no siRNA), Luc siRNA, or cycE siRNA, and then treated without (mock) or with indicated siRNA complex by intratumoral injection. Each complex contained 10 µg of siRNA (for mock, using PBS instead of siRNA) and 7.5 µl Oligofectamine (Invitrogen) in PBS, which was mixed according to manufacturer's instruction of Oligofectamine. These mice were treated weekly for 4 weeks, and sacrificed 7 days after the last treatment for tumor size comparison or sacrificed 2 days after the last treatment for anti-cycE immunohistochemistry or TUNEL assays. The tumor size was measured just before each treatment or after sacrifice, and the tumor volume was obtained by the following equation: V =  (length × width × height)/2 (V is the tumor volume). For assessment of the effectiveness of combination of cycE siRNA with doxorubicin (Dox), MDA-MB436 cells were injected into the second pair of mammary glands of the nude mice as described above (totally n = 75). Beginning on day 14 post the tumor cell injection, the mice with tumor burden were randomly divided into 5 groups (n = 15 for each group): mock (liposome alone), Luc siRNA, cycE siRNA, Dox (alone), and Dox + cycE siRNA. For the groups of mock, Luc siRNA or cycE siRNA, the mice were treated with liposome alone, Luc siRNA (10 µg), or cycE siRNA (10 µg), respectively, by weekly repeated intratumoral injection for 4 weeks. For the Dox alone group, the mice were only treated with Dox (2 mg/kg body weight) by weekly intraperitoneal injection over 4 weeks. For the Dox + cycE siRNA group, the mice were administered with Dox (2 mg/kg body weight) by weekly intraperitoneal injection following cycE siRNA treatment on the next day for 4 weeks. All of the above experiments were repeated two or three times.

### Immunohistochemistry

Tumors were dissected 2 days after the last treatment with mock or indicated siRNA, and then sectioned, deparaffinized, rehydrated, and stained with anti-cycE antibody according to manufacturer's instructions of Elite Universal ABC kit (Vector Laboratories). For each treatment group, at least two tumor samples, two slides per sample were analyzed. For each slide examined, 1000 cells were counted from 6 fields with 200X magnification and the percentage of cycE-positive cells, compared to total cells, were indicated on the y axis.

### TUNEL assay

Apoptotic cells were confirmed using the In Situ Cell Death Detection kit from Roche, following the manufacture's instruction. The apoptotic cells (purple staining) were counted under a microscope. The apoptotic index was defined by the percentage of purple cells among the total cells of each sample. For each cell line analyzed, 200 cells were counted from the fields with 200X magnification. As to each tumor sample analyzed, 1000 cells were counted from the fields with 200X magnification. The experiments were repeated at least three times.

### Synergistic analysis of cycE siRNA and Dox in cultured cells

SK-BR3 and MDA-MB436 cells (1×10^4^ cells/well) were transfected with different concentration of cycE siRNA. Twenty-four hours after transfection, the cells were split into 96-well culture plates and treated with different dosages of Dox for 8 days. The viable cells were then determined with MTT assay. For MTT assay, 25 µl MTT (3-[4,5-dimethylthiazol-2-yl]-2,5-diphenyltetrazolium bromide, Sigma) stock solution (5 µg/ml) was added to each well on the plate. Cells was incubated for 4 hours with MTT and then lysed in 100 µl of dimethylsulfoxide (DMSO). Conversion of MTT to formazan by metabolically viable cells was determined by microplate reader at 540 nm wavelength. The synergistic inhibitory effects were determined with combination index at IC80, IC90 and IC95 using the CI-isobologram method developed by Chou-Talalay [26], [27].

### Statistical analysis

Statistical analysis was carried out using a two-tailed Student's t-Test. Data were considered statistically significant at p value ≤0.05.

## Results

### CycE overexpression is suppressed in breast cancer cells by siRNA targeting

**Figure 1:**
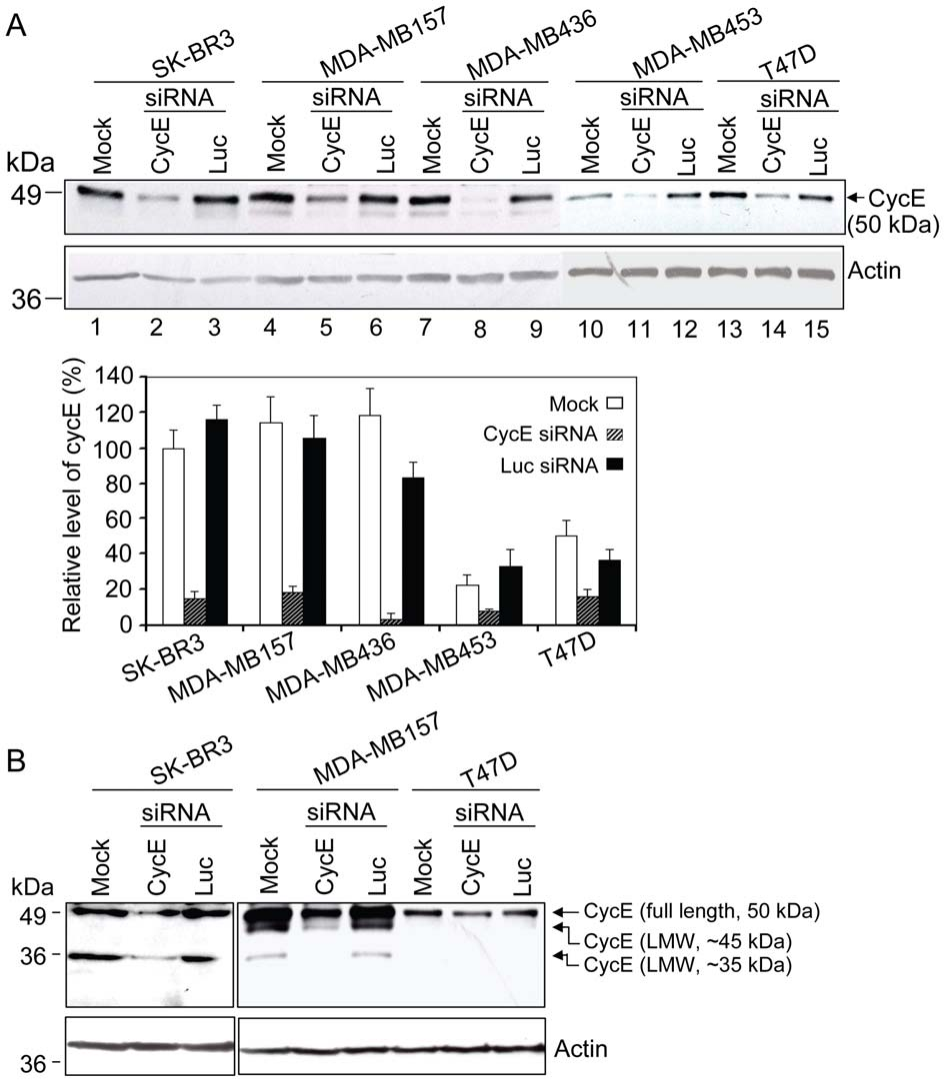
CycE siRNA is able to remarkably suppress cycE overexpression in breast cancer cell lines. **A**) Inhibition of cycE overexpression by cycE siRNA. Indicated cells were transfected with siRNA oligos targeting on either cycE or luciferase (Luc, control). Cells were harvested two days after transfection. The protein lysates were subjected to Western blotting analysis by using anti-cycE and anti-Actin antibodies, respectively (top panel). CycE protein levels (full length) from cycE or luc siRNA-treated cells were normalized by Actin. The normalized density of cycE in the mock SK-BR3 was arbitrarily set at 100%, and the relative level of cycE in other samples was obtained by comparing those normalized densities of cycE to that of cycE in the mock SK-BR3 (bottom panel). **B**) Suppression of the expression of LMW isoforms by cycE siRNA. The indicated cells were transfected with or without siRNA, and the whole lysates were collected and subjected to anti-CycE Western blot analysis. In SK-BR3 and MDA-MB157 cells, although LMW isoform pattern of cycE was different, the LMW isoforms as well as the full length cycE were inhibited by cycE siRNA. The LMW isoforms of cycE were undetectable in T47D.

To address if cycE can serve as a novel therapeutic target for breast cancer, we first used siRNA oligos to deplete cycE expression in breast cancer cells. The selected cycE siRNA targeted the mRNA sequence near the region encoding the cyclin box of cycE [23]. The siRNA oligos were transfected into three cycE-overexpressing breast cancer cell lines (SK-BR3, MDA-MB157 and MDA-MB436) and two cycE-low expressors (T47D and MDA-MB453). Protein levels of the full length cycE (50 kD) were reduced by cycE siRNA up to 96% in all five cell lines (Figure 1A, lanes 2, 5, 8, 11 and 14 of upper panel; Figure 1A, lower panel). LMW isoforms of cycE (∼45 kDa and ∼35 kDa) were significantly reduced as well (Figure 1B). The inhibitory effect of the cycE siRNA was shown to be specific since a control siRNA oligo targeting firefly luciferase mRNA (Luc) had no effect on cycE expression levels (Figure 1A). Moreover, siRNA oligos did not cause nonspecific downregulation of gene expression, as demonstrated by β-actin control (Figure 1A). These data indicate that cycE siRNA can effectively suppress the overexpression of both full length and LMW isoforms of cycE.

### Apoptosis and G1 arrest are induced in breast cancer cells by siRNA-mediated depletion of cycE overexpression

**Figure 2:**
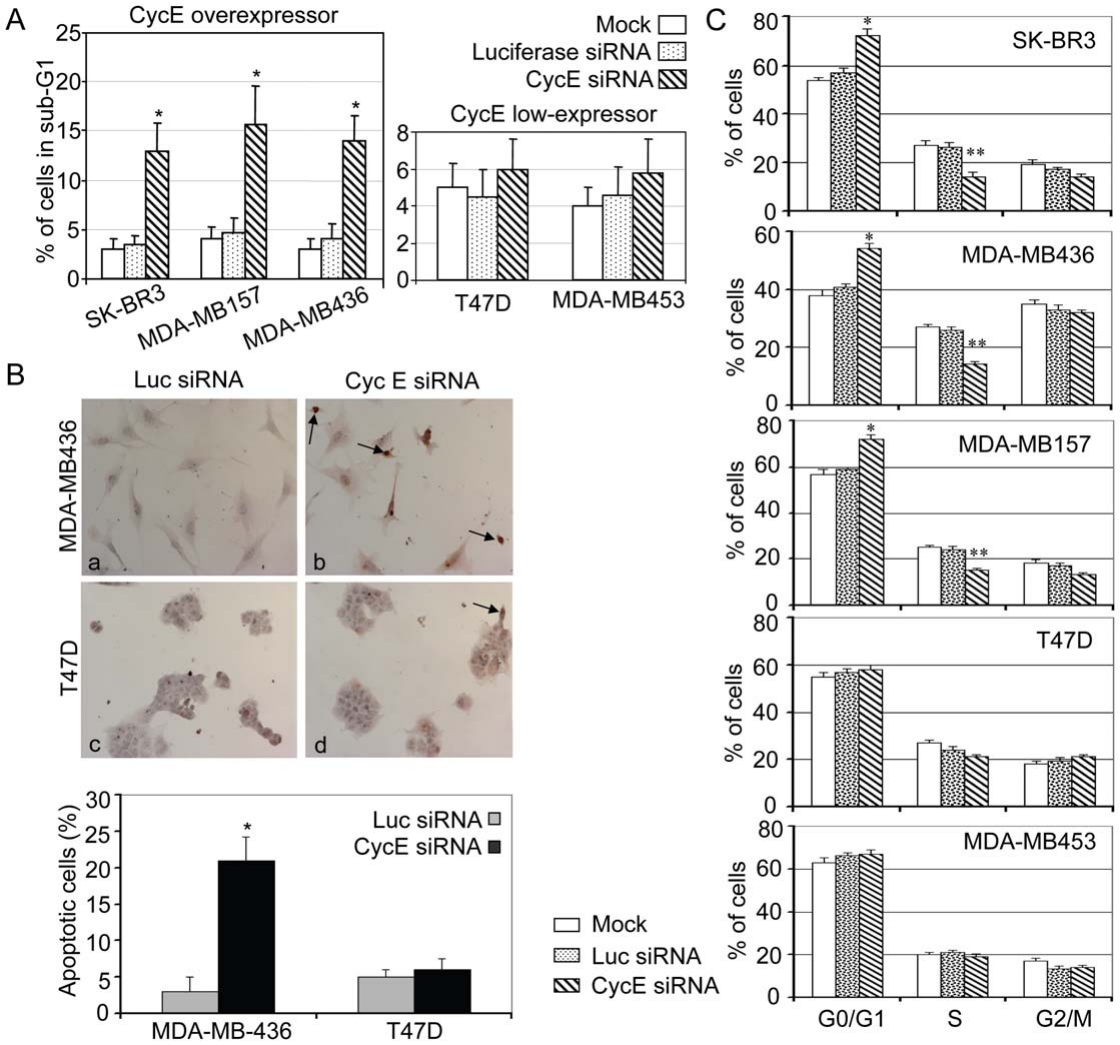
Apoptosis and decreased cell proliferation induced by depletion of cycE in cycE-overexpressing breast cancer cells. A) Downregulation of cycE promotes apoptosis of SK-BR3, MDA-MB436 and MDA-MB157, but not T47D and MDA-MB453. Ninety-six hours after siRNA transfection, the indicated adherent cells were collected by trypsinization and combined with cells floating in the medium. The apoptotic cells were then determined with flow cytometry. (*) p ≤0.05 compared with mock. B) Apoptosis in cycE-overexpressing cells are confirmed by TUNEL assay. Two days after siRNA transfection, the indicated cells were subjected to TUNEL analysis. Upper panel: apoptotic cells (purple staining) were detected in MDA-MB436, but not in T47D cells. Lower panel: Percentage of apoptotic cells in the above two breast cancer cells treated with cycE or control siRNA. C) S phase population is decreased by cycE siRNA in cycE-overexpressing cells. Treated cells were collected 48 h after transfection of cycE siRNA in three cycE-overexpressing cells (SK-BR3, MDA-MB436 and MDA-MB157) and two cycE-low expressors (T47D and MDA-MB453). The cell cycle pattern (G0/G1, S and G2/M) was also determined by flow cytometry. Three individual experiments were performed. (*) p≤0.05 compared with mock in G0/G1 population, (**) p≤0.05 compared with mock in S population.

Since cycE and its functional complex cycE-Cdk2 play pivotal roles in G1/S transition, we next examined pattern changes of the cell cycle distribution and apoptosis in cycE-depleted cells. To determine if depletion of cycE promotes tumor cell death, flow cytometry was performed after transfection of the siRNAs. The cells were analyzed at different time points (72 h and 96 h) post-transfection and significant sub-G1 (apoptotic) populations were observed at 96 h in cycE-overexpressing cells (SK-BR3, MDA-MB157 and MDA-MB436); about 14% of these cells underwent apoptosis after transfection of cycE siRNA (Figure 2A, left panel). In contrast, only ∼4% of the same cell lines underwent apoptosis in the mock or luciferase siRNA-treated groups (Figure 2A, right panel). The cycE-overexpressing cells shrank, rounded up and detached from plates three days after transfection of cycE siRNA while the control siRNA treated group remained attached on the dishes and showed normal morphology, also suggesting that apoptosis had occurred. In addition, we confirmed the cycE siRNA-induced apoptosis in cycE-overexpressing cells by TUNEL assay (Figure 2B, a & b). Notably, in contrast to the cycE-overexpressing cells, we did not observe significant apoptosis in cycE-low expressing cells (MDA-MB453 and T47D) at 96 h after transfection (Figure 2A, right panel and Figure 2B, c & d), although the cycE protein level in these cells was effectively suppressed by cycE siRNA (Figure 1). These data reveal that depletion of cycE specifically triggers apoptosis in the cycE-overexpressing cells.

Additionally, 48 h after cycE siRNA transfection, we observed increased G0/G1 and decreased S phase population in all three cycE-overexpressing cell lines tested but not in cycE-low expressing cells MDA-MB453 or T47D (Figure 2C). Thus, these results together indicate that cycE siRNA exhibits a specific inhibitory effect on cycE-overexpressing breast cancer through promotion of apoptosis as well as inhibition of G1/S phase transition.

### Depletion of cycE inhibits proliferation and transformation in the cycE-overexpressing breast cancer cells

**Figure 3:**
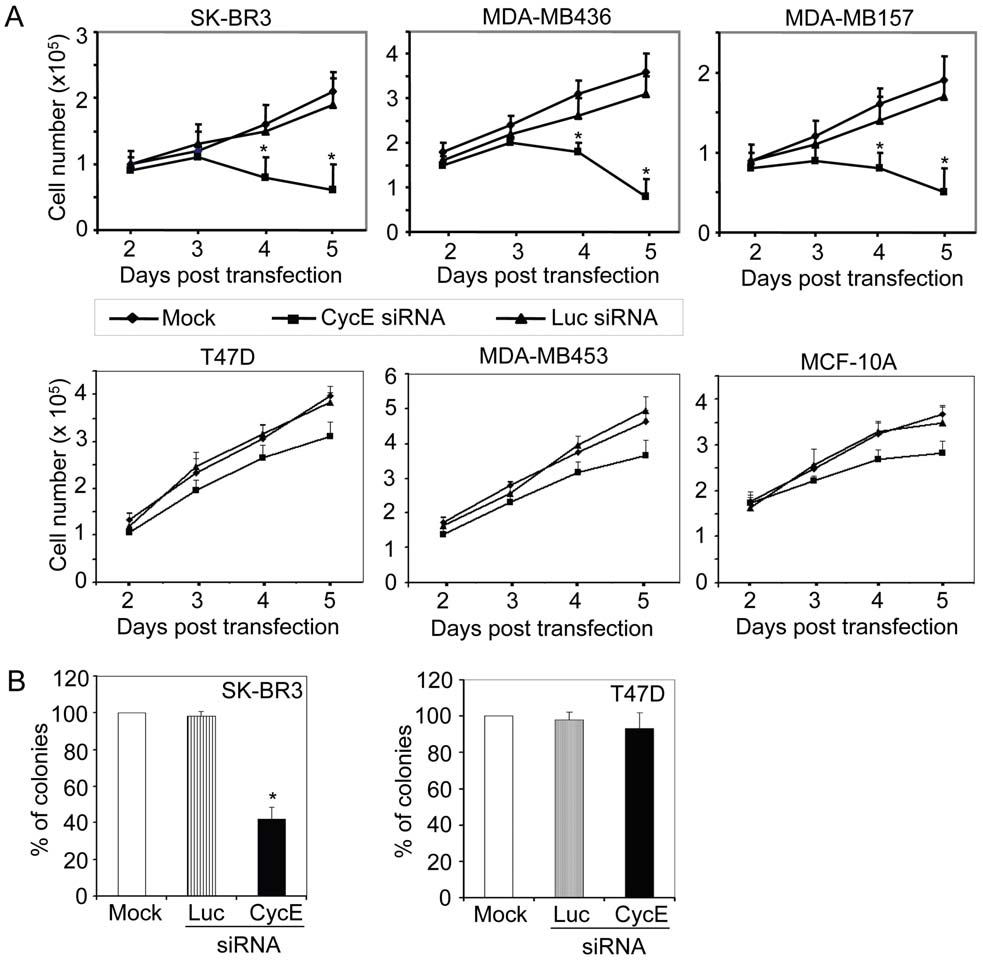
Inhibition of cell transformation by cycE siRNA *in vitro.* **A**) Growth curves of breast cancer and normal epithelial cells in response to cycE siRNA. The viable cells were counted at the indicated time points after transfection of siRNA oligos. The data shown here represent the averages from three independent experiments. **B**) Suppression of colony formation in soft agar by cycE siRNA. The indicated cells were transfected with siRNA targeting on cyclin E (cycE) or luciferase (Luc) and then seeded in 0.35% agar containing DMEM with 10% FBS. The cells without any oligo transfection (mock) were used as a control. The cell viability for each group (mock, Luc, or cycE) was determined by colony formation assay. The number of colonies on soft agar was counted 3-5 weeks later and was normalized by their cell viability. The normalized number of colonies on soft agar for the groups of Luc or cycE was standardized against the control cells (mock, set at 100%) and indicated on the y axis. The data were the averages from two independent triplicate experiments. (*) p≤0.001 compared with mock.

To determine whether cycE siRNA actually affects proliferation of breast cancer cells, we examined the growth curves of three cycE-overexpressing cell lines and two cycE-low expressors in response to cycE siRNA treatment. As shown in Figure 3A, cycE siRNA significantly decreased the cell number of all three cycE-overexpressing cell lines (SK-BR3, MDA-MB157 and MDA-MB436) as compared to the control. However, for the cycE-low expressing cells (T47D and MDA-MB453) or normal breast epithelial cells (MCF10A), the cell number was not attenuated dramatically after cycE siRNA transfection (Figure 3A). CycE depletion also inhibited the transformation phenotype of cycE-overexpressing cells. As shown in the Figure 3B and Figure S1, cycE siRNA significantly reduced the ability of SK-BR3 to grow on soft agar, a well known assay that measures cell transformation. The transformation-suppressing activity was also observed in MDA-MB157 and MDA-MB436 cell lines when we depleted their cycE's expression (data not shown). In contrast, we did not observe significant transformation inhibitory effects on T47D (Figure 3B and Figure S1) and MDA-MB453 (data not shown) by cycE siRNA targeting. Taken together, cycE siRNA can dramatically inhibit proliferation and transformation of the cycE-overexpressing breast cancer cells.

### CycE siRNA treatment is able to suppress tumor growth *in vivo*

**Figure 4:**
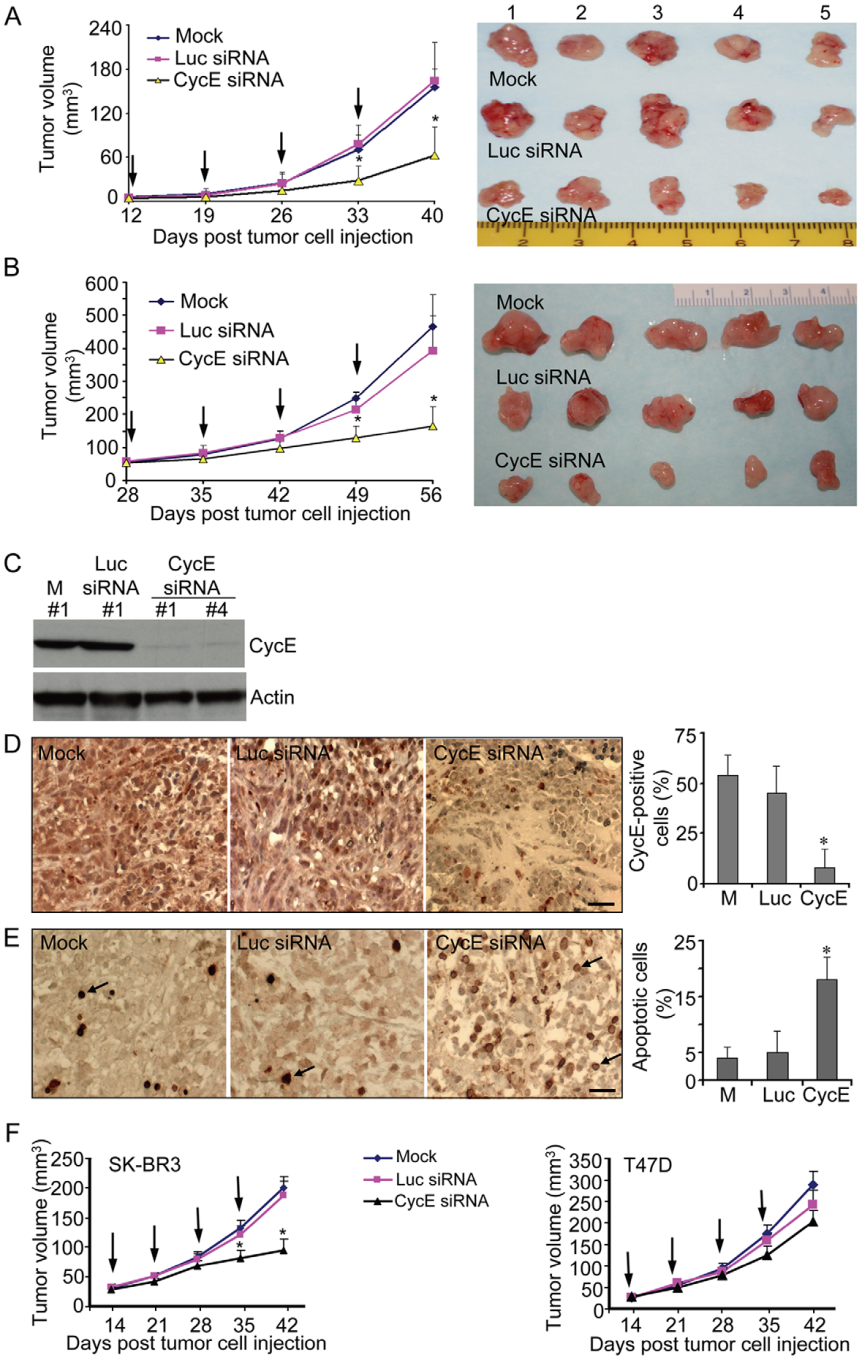
Tumor growth *in vivo* is dramatically inhibited by cycE siRNA. **A**) The growth of MDA-MB436 tumor xenografts is suppressed by cycE siRNA treatment. The MDA-MB436 cells were injected into the mammary glands of the nude mice. At around 2 weeks after tumor cell injection, the mice with tumor burden were randomly grouped (n = 15 for each group) and treated weekly by intumoral injection over 4 weeks with indicated siRNA (10 µg) or liposome alone (mock). Arrows indicated the days when mice were administered. Tumor size was measured weekly before each administration. The treated mice were sacrificed at day 40 post tumor cell injection and tumor samples were collected. Representative tumor samples from each indicated treatment group were shown in the right panel and the tumors from cycE-siRNA treated groups were much smaller as compared to mock or Luc-siRNA groups. (*) p≤0.05 compared with mock. Scale unit: 1 cm. **B**) Growth inhibition of the established tumor xenografts (∼50 mm^3^) by cycE siRNA. The MDA-MB436 cells were injected into the mammary glands of the nude mice. When the tumor size reached to ∼ 50 mm^3^ at around 4 weeks after tumor cell injection, the mice were randomly grouped (n = 15 for each group) and treated weekly by intratumoral injection for 4 weeks without (mock) or with indicated siRNA (10 µg). Arrows indicated the days when mice were administered. The treated mice were sacrificed at day 56 post tumor cell injection and tumor samples were collected. Representative tumor samples from each indicated treatment group were shown in the right panel. (*) p≤0.05 compared with mock. Scale unit: 1 cm. **C**) Assessment of reduced cycE protein levels in cycE siRNA-treated tumors. MDA-MB436 tumors from indicated treatment groups were collected 2 days after the last treatment with mock (M) or indicated siRNA and analyzed for cycE protein levels by Western blots using anti-cycE or anti-actin antibodies. Here shown are the representative results from the indicated samples of each group in **A**. M: Mock. **D**) Confirmation of reduced CycE expression in response to cycE siRNA treatment by immunostaining. MDA-MB436 tumors from indicated treatment groups were sectioned and analyzed for cycE expression using anti-cycE immunohistochemical staining (left panels). For each tumor sample, 1000 cells were counted, and the cycE-positive cells were summarized in the right panel. (*) p≤0.01 compared with mock (M). Scale bar, 25 µm. **E**) Apoptosis is induced in the cycE siRNA-treated tumors. Apoptosis was examined by TUNEL assay in MDA-MB436 tumor sections with indicated treatment. For each tumor sample, 1000 cells were counted, and the apoptotic cells were summarized in the right panel. (*) p≤0.01 compared with mock (M). Black arrows, apoptotic cells. Scale bar, 25 µm. **F**) CycE siRNA treatment dramatically inhibits tumor growth of SK-BR3 but not the growth of T47D in mice. SK-BR3 cells or T47D cells were injected into the mammary glands of the nude mice, respectively. On day 14 after implantation of tumor cells, the mice were treated weekly for 4 weeks with indicated siRNA (10 µg) or liposome alone (mock) (n = 15 for each group). Arrows indicated the days when mice were administered. Tumor size was measured weekly before each administration. (*) p≤0.05 compared with mock.

To determine whether cycE siRNA could suppress breast cancer growth *in vivo*, we established breast tumors in nude mice and then treated them with cycE siRNA. MDA-MB436 cells can easily grow tumors in nude mice and, thus, we mainly selected this cycE-overexpressing cell line to establish the tumor model in the mammary glands of the nude mice. MDA-MB436 cells were injected into the mouse mammary glands (two injected points per mouse). About two weeks later, the nude mice with tumor burden (n = 15 for each group) were treated with indicated siRNA complex or liposome alone (mock) by intratumoral injection. CycE or control siRNA treatment was administered weekly for 4 weeks. As shown in Figure 4A, we found that tumor growth in the cycE siRNA group was dramatically inhibited as compared to the controls. To further assess if cycE siRNA was able to suppress the tumor growth, we did not treat the mice with the siRNA until the tumor burden in each site reached over ∼50 mm^3^ at around 4 weeks after tumor cell injection. After 4 weeks treatment, the tumor progression from the cycE siRNA treatment group was significantly suppressed (Figure 4B), which demonstrated cycE siRNA's inhibitory ability on the established tumors. In addition, Western blots and immunohistochemistry analysis showed that cycE protein levels were also decreased in most of the cycE siRNA-treated tumors (Figure 4, C & D). Indeed, we observed much fewer cells with cycE overexpression in tumors treated with cycE siRNA as compared to those treated with PBS or control siRNA (Luc siRNA) (Figure 4D, cycE siRNA panel). Furthermore, TUNEL assay showed that more apoptotic cells were in the cycE siRNA-treated tumors (Figure 4E), indicating that cycE depletion was able to induce apoptosis *in vivo* and as a result, led to tumor suppression. We also tested the inhibitory efficacy of cycE siRNA on another cycE-overexpressing line, SK-BR3 as well as T47D, a cycE-low expressing line by tumorigenecity assay. We found that treatment of cycE siRNA could dramatically inhibit the tumor growth of SK-BR3 while this treatment exerted only mild suppressing effect on T47D growth in mice (Figure 4F). Taken together, treatment of cycE siRNA can inhibit cycE-overexpressing breast tumor growth *in vivo*, indicating that cycE siRNA may serve as a novel therapeutic agent for treating breast cancer with cycE overexpression.

### Synergistic inhibitory effects are achieved by combination of cycE siRNA and Dox

**Figure 5:**
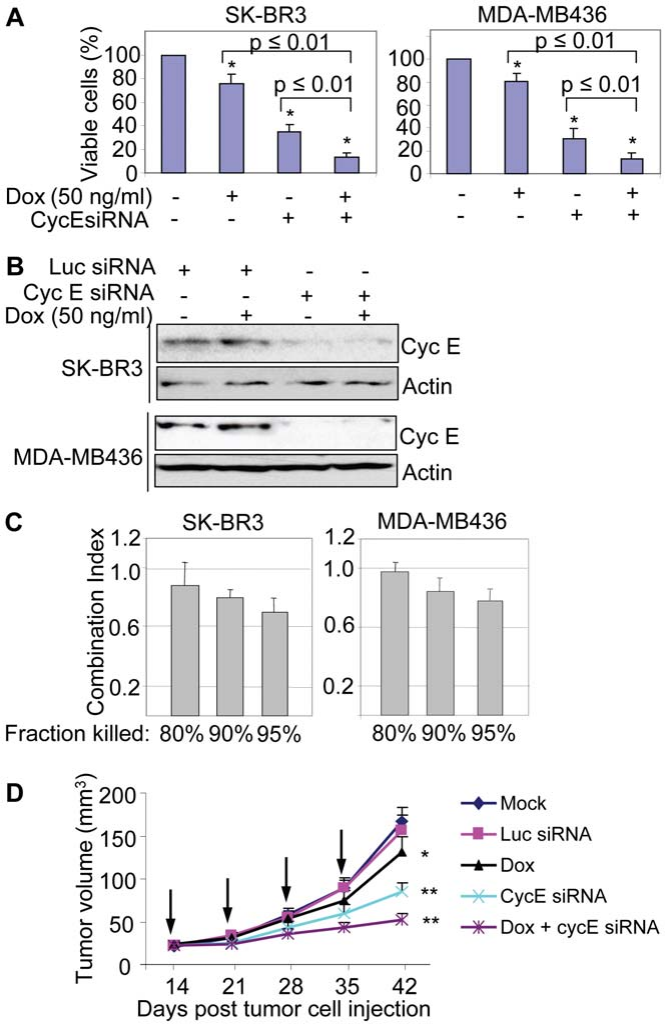
Enhanced inhibitory effects by combination of cyc E siRNA with Dox. **A**) Sensitization of breast cancer cells to Dox by treatment of cyc E siRNA. Indicated breast cancer cells (1×10^5^/well in 6-well plates) were transfected with cyc E siRNA (0.1 µg/well) for 24 hours. The cells were then split into 96-well plates (2×10^3^ cells/well) and treated with Dox (50 ng/ml) for 8 days. MTT assay was performed to determine cell viability. Y-axis represented the relative MTT value from each treatment. (*) p≤0.05 compared with mock. **B**) CycE levels are not affected by Dox. CycE-overexpressing breast cancer cells (SK-BR3 and MDA-MB436) were treated without or with siRNA alone, Dox (50 ng/ml) alone or in combination for 48 h. Treated cells were collected and the lysates were subjected to Western blot analysis with anti-cycE and anti-Actin antibodies, respectively. **C**) Synergistic cytotoxicity by combination of cyc E siRNA with Dox. SK-BR3 and MDA-MB436 cells were treated with different concentration of siRNA (0–0.1 µg/well in six-well plates) and split into 96-well plates. After 8 days incubation with different concentration of Dox (0–100 ng/ml), cytotoxicity was determined by MTT assay. The CIs were calculated using the CI-isobologram method by Chou-Talalay. CI = 1 indicates an additive effect; CI<1 indicates synergism; CI>1 indicates antagonism. Data plotted are the CI value at 80, 90 and 95% fraction killed and are means of three independent experiments; bars, SD. **D**) CycE siRNA in combination with Dox is able to effectively inhibit the breast tumor growth in mice. MDA-MB436 cells were injected into the mammary glands of the nude mice. Beginning on day 14 post tumor cell injection, the mice were weekly treated with cycE siRNA (10 µg/injection), luciferase siRNA (Luc-siRNA), liposome alone (mock), Dox alone (2 mg/kg body weight via intraperitoneal injection) or cycE siRNA + Dox over 4 weeks as described in Methods section. Arrows indicated the days when mice were administered. The effects of treatment on tumor growth were determined by weekly measuring tumor volume. Dox: doxorubicin. (*) p≤0.05 and (**) p≤0.01 compared with mock (M) after 4 administrations.

To broaden the potential clinical applications, we tested if there was any synergistic effect on breast cancer cell growth by combination of cycE siRNA with Dox (50 ng/ml), a chemotherapeutic drug commonly used to treat breast cancer patients [28], [29]. SK-BR3 and MDA-MB436 cells were treated with cycE siRNA or Dox alone, or in combination. We found that combination of cycE siRNA with Dox exhibited a much stronger cell-killing effect than cycE siRNA alone or Dox alone in both cell lines (Figure 5A), while Dox at 50 ng/ml had no apparent effects on expression level of cycE in SK-BR3 and MDA-MB436 (Figure 5B). To determine if this inhibitory effect was synergistic, we calculated the combination indexes (CI) using the CI-isobologram method developed by Chou-Talalay [26], [27]. As shown in Figure 5C, CIs at IC90 and IC95 were both less than 1 for SK-BR3 and MDA-MB436 cells, indicating there was synergistically inhibitory effect on these cancer cells by combination of the cycE siRNA with Dox. In addition, we examined the therapeutic effect of this combinational treatment in mice. About 14 days after inoculation of MDA-MB436 cells, the animals were divided into 5 groups (n = 15 for each group). For the groups of mock, Luc siRNA and cycE siRNA, the mice were treated with liposome alone (mock), Luc siRNA, and cycE siRNA, respectively, by weekly repeated intratumoral injection for 4 weeks. For the Dox alone group, the mice were treated with Dox by weekly intraperitoneal injection for 4 weeks, and for the Dox + cycE siRNA group, the mice were administered with Dox by weekly intraperitoneal injection following cycE siRNA treatment on the next day for 4 weeks. We found that administration of cycE siRNA combined with Dox was able to suppress tumor growth of MDA-MB436 with a much stronger effect than cycE siRNA oligos alone or Dox alone (Figure 5D). Together, combination of cycE siRNA with Dox may provide a novel treatment option for breast cancer with cycE overexpression.

## Discussion

CycE is aberrantly expressed in many types of cancer [14], [30]–[32]. Indeed, high levels of the wild-type and LMW isoforms of cycE are associated with poor prognosis of breast cancer patients [14]. Importantly, cycE overexpression is also correlated with triple-negative (ER-, PR-, and HER2-negative) basal-like breast cancers [33] which lack specific therapeutic targets. In this study, we used cycE siRNA to target cycE overexpression and assessed its ability to suppress breast cancer growth in nude mice. Our results revealed that cycE siRNA effectively inhibited the cycE overexpression of both full length and LMW isoforms (Figure 1). Depletion of cycE promoted apoptosis of cycE-overexpressing cells and blocked their proliferation and transformation phenotype (Figures 2 and 3). We also showed that cycE siRNA inhibited breast tumor growth in nude mice (Figure 4). In addition, we found that cycE siRNA synergistically enhanced the cell killing effects of Dox in cell culture and this combination greatly suppressed tumor growth in mice (Figure 5). Thus, our study clearly demonstrates the therapeutic potential of cycE siRNA for treating cycE-overexpressing breast cancer, including triple-negative breast cancer. Our results also indicate that cycE, which is overexpressed in 30% of breast cancer, may serve as a novel and effective therapeutic target.

Although the full length cycE is usually expressed in both normal and tumor cells, the LMW cycE isoforms are predominantly expressed in tumor cells. These LMW isoforms of cycE are mainly post-translationally processed by proteinase (e.g. elastase) in tumor cells to produce two sets of doublets with subsequent modifications when observed in Western blots (33–35 kDa, and 44–45 kDa) [13], [34]. LMW cycE isoforms function in the same manner as the full length proteins, and show increased binding efficiency to its partner kinase CDK2, leading to accelerated entry to S phase and genomic instability [35], [36]. Importantly, as we demonstrated above, our cycE siRNA effectively inhibited not only expression of the full length but also LMW isoforms of cycE proteins, which, in turn, led to suppression of cell growth and tumorigenesis. All these results indicate that cycE siRNA treatment can reverse the malignancy of breast cancer induced by overexpression of both wild-type and LMW isoforms of cycE.

High level of cycE correlates with triple-negative breast cancers [33]. Indeed, two of three cycE-overexpressing cell lines that were used in this study (MDA-MB436 and MDA-MB157) are triple-negative cancer cells [24]. Notably, cycE siRNA alone (Figures 3 and 4) or in combination with Dox (Figure 5) can effectively inhibit the growth of these cancer cells both *in vitro* and in mice. Thus, this combination therapy may serve as an effective treatment option for triple-negative breast cancers since there are no specific treatment guidelines for the triple-negative cancers, which appear to be very metastatic and have a poor prognostic outcome.

Our results demonstrate that cycE siRNA exerts robust anti-tumor activity via promoting apoptosis and inhibition of DNA replication. The induced apoptosis is only observed in cycE-overexpressing cells (SK-BR3, MDA-MB157 and MDA-MB436) but not in cycE-low expressing cells (MDA-MB453 and T47D), and this specificity should increase the therapeutic index of siRNA-based therapies for cycE-overexpressing cancers. It is unclear how cycE depletion triggers apoptosis in cycE-overexpressing cells. However, it is well known that tumor cells are highly dependent on an activated oncogene for their survival and/or proliferation, a phenomenon called “oncogene addiction” [37]. Inactivation of these activated oncogenes leads to apoptosis and anti-tumor functions. The most convincing evidence for the concept of oncogene addiction comes from the examples of therapeutic efficacy of antibodies or drugs that target specific oncogenes in human cancers such as the antibody trastuzumab (Herceptin), which targets the receptor tyrosine kinase HER-2/NEU in breast cancer. Our studies here support that cycE-overexpressing cancers are cycE addiction, i.e., highly rely on the activity of cycE for continued cell proliferation and survival. Importantly, in addition to promotion of apoptosis, depletion of cycE by its siRNA sensitizes the cell killing effect of Dox in the cycE-overexpressing breast cancer cells. Dox is a chemodrug targeting S-phase cells via inhibition of the topoisomerase II (TOP2) [38]. Our flow cytometry data show that depletion of cyc E does not lead to accumulation of cells in S phase, suggesting that sensitization to Dox is directed through mechanisms other than S-phase accumulation. Interestingly, it has been shown that the cell survival pathways such as NF-êB, Akt and Bcl-2 family can be activated to antagonize the cytotoxic effects of Dox [39]-[41]. Also, blockage of these pathways sensitizes breast cancer cells to Dox [39]. Therefore, in the future, it will be interesting to investigate if cycE overexpression may enhance the survival pathways and therefore increase the resistance to Dox.

A potential concern for siRNA therapy is the delivery effectiveness of siRNA *in vivo*. In this study, cycE siRNA was delivered using liposome by intratumoral injection and we demonstrated this regime being sufficient to inhibit cycE expression *in vivo* and as a result, led to tumor suppression. CycE siRNA used here for treatment is a non-chemically-modified siRNA, which has relatively limited *in vivo* stability and membrane permeability. To apply cycE siRNA therapy in clinic in the future, it will be greatly beneficial if we can modify this siRNA to reduce its sensitivity to nucleases and increase its cellular uptake. Recently, various approaches have been adapted to chemically modify the siRNAs to increase its nuclease resistance as well as its intracellular uptake [17], [42], [43]. Thus, it will be of great interest to test if chemical modifications of our cycE siRNA will improve its therapeutic efficacy, especially for systematic treatment in mice. In addition to chemical modification of siRNA, novel delivery systems can be employed to improve stability and efficacy of cycE siRNA [17], such as carrying siRNA by targeted nanoparticles to further improve the therapeutic efficacy of cycE siRNA in the future.

## Supporting Information

Figure S1 Cyclin E siRNA inhibits colony formation in soft agar in cyclin E-overexpressing cells, but not in cyclin E-low expressing cells. Here shown are the representative data of colony formation in soft agar from SK-BR3 (top panel) and T47D (bottom panel). The viability of each group (%) was monitored as described in Methods section, and indicated at the bottom of each panel.
(9.73 MB TIF)
