# Supplementary figures and images for: siRNA-Based Targeting of Cyclin E Overexpression Inhibits Breast Cancer Cell Growth and Suppresses Tumor Development in Breast Cancer Mouse Model
Source: PLoS One. 2010 Sep 20;5(9):e12860. doi: 10.1371/journal.pone.0012860 (PMC2942895; doi:10.1371/journal.pone.0012860)

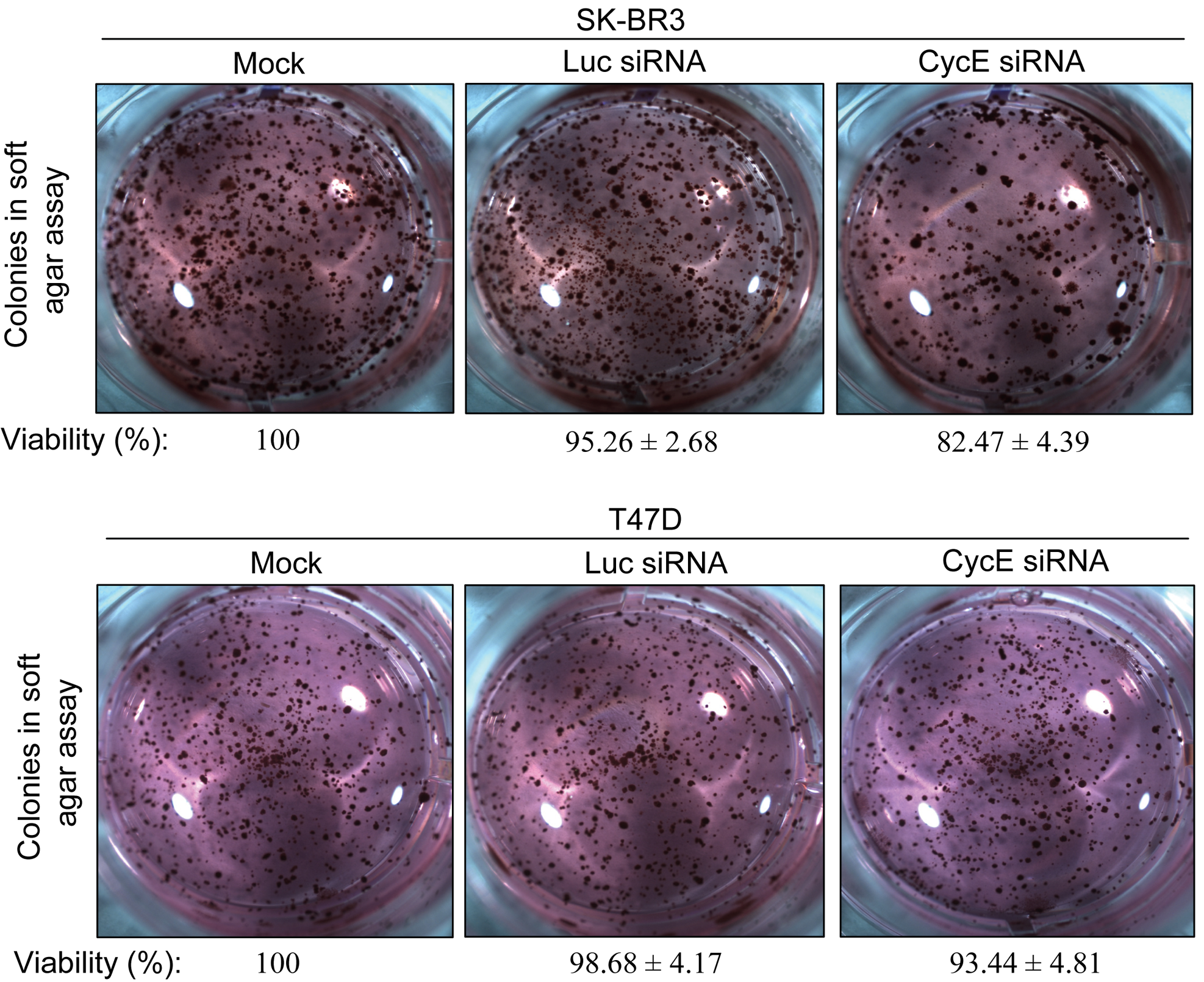

Supplement: Figure S1 — Cyclin E siRNA inhibits colony formation in soft agar in cyclin E-overexpressing cells, but not in cyclin E-low expressing cells. Here shown are the representative data of colony formation in soft agar from SK-BR3 (top panel) and T47D (bottom panel). The viability of each group (%) was monitored as described in Methods section, and indicated at the bottom of each panel. (9.73 MB TIF) [file pone.0012860.s001.tif]
